# How is COVID-19 perceived by medical students? A survey in Aceh province, Indonesia

**DOI:** 10.4102/jamba.v13i1.1146

**Published:** 2021-11-19

**Authors:** Rina S. Oktari, Muhammad D. Detiro, Safrizal Rahman

**Affiliations:** 1 Department of Family Medicine, Faculty of Medicine, Universitas Syiah Kuala, Banda Aceh, Indonesia; 2 Department of Surgery, Faculty of Medicine, Universitas Syiah Kuala, Banda Aceh, Indonesia

**Keywords:** COVID-19, pandemic, disaster, knowledge, attitude, preventive behaviour

## Abstract

The coronavirus disease 2019 (COVID-19) has burdened the health system and medical education programmes both locally and globally, requiring medical students to continue their education whilst engaging in prevention programmes to support primary health services. This study aims to describe medical students’ knowledge, attitudes in the prevention of COVID-19, and to determine the relationship between the knowledge of COVID-19 preventive behaviour and attitudes towards it. This study used an analytical cross-sectional observational design with a sample of 290 students. Data were collected through a self-assessment method using a validated questionnaire. Analysis of the frequency distribution test for knowledge found that 54.1% of the respondents and 99.0% of the students had a good understanding of the mode of transmission of COVID-19. The majority of medical students (73.1%) also had a good attitude towards COVID-19 and around 84.3% of the students considered that good ethics is needed when coughing or sneezing during this pandemic. As many as 46.6% of the respondents had good preventive behaviour against COVID-19 and always imposed restrictions on using public transportation during a pandemic. The Spearman’s correlation test shows a weak but meaningful relationship between knowledge (*r* = 0.214, *p* = 0.000) and COVID-19 preventive behaviour, and a significant relationship between attitudes (*r* = 0.477, *p* = 0.000) towards COVID-19 preventive behaviour. This study concludes that medical students have good knowledge, preventive behaviour and an understanding of infection risk. An enhanced knowledge and awareness will increase preventive behaviours that will provide support in controlling the disease.

## Introduction

Severe acute respiratory syndrome coronavirus 2 (SARS-COV-2) is a new type of virus that causes coronavirus disease 2019 (COVID-19) (Huang & Zhao 2020). This disease was first reported at the end of December 2019 in Wuhan, China (Rothan & Byrareddy 2020). The World Health Organization (WHO) has declared COVID-19 as a public health emergency. The WHO has also designated this virus as a pandemic disease. This is because the level of spread of the virus is very high. Although research states that the spread of this virus originated in animals, it is mostly spread from humans to other humans (Rezaeetalab et al. 2020; WHO 2020a).

COVID-19 spreads rapidly; and has globally spread from China to more than 100 countries in a period of less than two months. The explosion of COVID-19 cases worldwide occurred in February 2020, with more than 82 000 positive cases and 2800 deaths. Chinese Center for Disease Control and Prevention (China CDC) reported that about 95% of the positive cases and 97% of these deaths are from China. As of 16 December 2020, the worldwide COVID-19 pandemic figures reached 18 986 629 cases and 712 334 deaths; and they still continue to increase daily and the virus has spread throughout all countries of the world without exception (De Ceukelaire & Bodini 2020; Rezaeetalab et al. 2020).

The COVID-19 cases in Indonesia have increased significantly since the first detection. The COVID-19 Task Force in Indonesia found an increase of nearly 6,000 positive cases per day after the government conducted a number of mass tests throughout Indonesia. The first COVID-19 case was detected in Indonesia on 02 March 2020. Two patients tested positive after a series of examinations were carried out. The number of positive patients continued to increase after contact tracking was carried out on COVID-19 sufferers. To date, more than 600 000 people in Indonesia have been infected with the virus, and more than 18 000 people have died from it (WHO 2020b).

Aceh Province (on the northwest tip of Sumatra Island) is one of the Indonesian provinces affected by COVID-19 and cases continue to increase and to date more than 6000 people have contracted COVID-19 in this province (Dinas Kesehatan Provinsi Aceh 2020). The increase in cases will certainly continue to burden the health system and medical education programmes locally and globally, which require medical students to be able to continue with their education and also carry out prevention programmes to support primary health services. This needs to be undertaken so that the people of Aceh Province who still have poor knowledge, attitudes and behaviour regarding the prevention of COVID-19 can continue to improve their prevention behaviour through a programme of education and motivation. Because a person’s knowledge and attitude have an important role in the behaviour required for preventing COVID-19, it is also noteworthy that in theory a person’s positive attitude and knowledge can directly influence how individuals behave in a proper manner regarding the prevention of this disease. Importantly, if someone has a low level of knowledge and a bad attitude towards this virus, it will have a negative impact on that person’s behaviour in complying with the COVID-19 prevention protocols. Therefore, it is necessary to utilise the role of health workers, such as medical students, to continue educating the public, without exception, with the goal of preventing the continuing spread of this virus (Kapoor 2016; Kim et al. 2020a; Saefi et al. 2020).

Based on the aforementioned, this study aimed at assessing how medical students in Aceh perceive COVID-19. This is because medical students who are part of the society also have an important role in disseminating information about prevention against COVID-19. Therefore, if medical students have poor knowledge and attitudes about this virus, it will have an impact on preventive behaviour carried out by the community itself. This study aimed at determining: (1) the knowledge, attitudes and preventive behaviours possessed by medical students, and (2) whether there is any influence of the existing knowledge and attitudes of medical students on their own behaviour regarding COVID-19 prevention programmes.

## Research methods

### Research design and population

This research is an analytic observational study that seeks to know the relationship between variables by analysing the data that have been obtained. This research used a cross-sectional design to determine the relationship between knowledge and attitudes towards COVID-19 prevention behaviour amongst medical students in Aceh Province, Indonesia. The study was conducted in Faculties of Medicine at three universities in the province of Aceh, Indonesia: (1) Universitas Syiah Kuala (USK), (2) Universitas Malikussaleh (UNIMAL), and (3) Universitas Abulyatama (UNAYA) with research time from April to August 2020.

The participants in this study were all medical students of the Faculties of Medicine in Aceh Province from the three above mentioned universities with a total student population of 1032 students. The data were taken from the Faculty of Medicine of each university with a sample of all students who met the inclusion criteria and did not meet the exclusion criteria.

### Inclusion and exclusion criteria

The inclusion criteria in this study were medical students studying at universities in Aceh who were willing to become participants by filling out the e-questionnaire provided. Meanwhile, the exclusion criteria were medical students who had filled out the given e-questionnaire, but the questionnaire was either invalid or incomplete.

### Sampling technique

The sampling technique in this study was stratified random sampling, by dividing a population into strata, selecting a simple random sample from each stratum, then combining it into a sample to determine the population parameters (Elfil & Negida 2019; Salkind 2010). After using the Slovin’s formula, it was calculated that this study required a sample of 290 Medical Faculty students in Aceh Province. Furthermore, the total sample was again divided using proportional sampling formula to determine how many students were sampled from each university:

$$n=\frac{\Sigma \text{ total of medical students in the university}}{\Sigma \text{ total population}}\times \text{minimum sample}$$ [Eqn 1]

As a result, the number of samples obtained is detailed in Table 1.

**TABLE 1:** Sample selection.

Location	Total of medical students	Total sample
Universitas Syiah Kuala	478	134
Universitas Abulyatama	277	78
Universitas Malikulsaleh	277	78
**Total**	**1032**	**290**

### Validity

In knowing a relationship between knowledge and attitudes with prevention behaviour against COVID-19, a research instrument is needed in the form of an online questionnaire (e-questionnaire) that has been tested for validity and reliability. The definition of validity according to the Journal of Graduate Medical Education (Accreditation Council for Graduate Medical Education, Chicago, Illinois, USA) is to show the degree of accuracy between the actual data occurring on the object of study and the data collected by the researcher (Wright et al. 2016). Whilst the reliability test has the function to determine whether the questionnaire used in this study shows the level of accuracy and consistency even though this questionnaire is used two or more times. The reliability test is carried out to assess how much a parameter can be trusted, where the reliability test always gives the same product even though the test is repeated many times.

In this study, researchers adopted and modified the questionnaire that had been developed by Bhagavathula et al. (2020) and a Behaviour Index issued by the Central Bureau of Statistics of the Republic of Indonesia to be used as material for the questionnaire of the present study.

The validity test of the questionnaire uses expert opinion (expert judgement). Seventy percent of the experts who validated this study were female and also all the experts (100%) were lecturers in the medical faculty who also played roles in dealing with COVID-19 in Aceh province and the disaster as a whole. The material of the research instrument itself has also been measured using the content validity ratio (CVR) as an assessment material for a validated questionnaire (with score > 0.79).

### Statistical analysis

Statistical analysis in this study was measured using the Statistical Package for the Social Sciences (SPSS version 20.0) programme. Frequency distribution tabulations were used to describe each variable and assess the description of knowledge, attitudes and preventive behaviour of medical students in order to test the univariate analysis. Meanwhile, for the bivariate analysis, the researcher used the Spearman’s correlation test to determine the strength of the relationship between the variables.

## Result

This research was conducted with a total population of 1032 students from which 290 respondents managed to meet the inclusion criteria. As aforementioned, the respondents were obtained from three different universities in the same province.

### Demographic characteristics of research subjects

Demographic characteristics of the participants in this study can be seen in Table 2. As stated above, the majority of respondents were female (74.8%). Most of the medical students were aged 19–21 years (85.9%) and were from USK (46.2%).

**TABLE 2:** Demographic characteristics of respondents (*n* = 290).

Characteristics	Frequency	Percentage (%)
**Gender**		
Male	73	25.2
Female	217	74.8
**Age (years)**		
18	29	10.0
19	86	29.7
20	104	35.9
21	59	20.3
> 21	12	2.1
**University**		
Universitas Syiah Kuala	78	26.9
Universitas Malikussaleh	78	26.9
Universitas Abulyatama	134	46.2

### Knowledge, attitudes and preventive behaviour of medical students against COVID-19

Measurement of knowledge, attitude and practices (KAP) regarding COVID-19 amongst medical students in Aceh Province were assessed using a questionnaire that the researchers had compiled by asking for expert opinions and then validating them. From the analysis (see Table 3), the majority of medical students in Aceh Province had good knowledge (54.1%) and attitudes (73.1%) towards COVID-19. Most of the respondents (46.6%) also performed good preventive behaviour and adhered to health protocols.

**TABLE 3:** Demographic characteristics of respondents (*n* = 290).

Characteristics	Frequency	Percentage (%)
**Knowledge**		
Less	17	5.90
Moderate	116	40.00
Good	157	54.10
**Attitude**		
Less	10	3.40
Moderate	68	23.40
Good	212	73.10
**Behaviour**		
Less	26	9.00
Moderate	129	44.50
Good	135	46.60

Table 4 presents the distribution of respondents’ answers related to knowledge towards COVID-19. Most medical students could answer correctly all the statements given and only a few answered incorrectly. However, the most correctly answered statement was about knowing the meaning of COVID-19 and its abbreviation (acronym), where 99.7% of students answered this statement correctly. Meanwhile, in the statement related to the incubation period of COVID-19, as many as 33.4% of students answered incorrectly.

**TABLE 4:** Respondents’ answers related to knowledge of COVID-19 (*n* = 290).

Statement	Respondents with correct answer	
	Frequency	Percentage (%)
COVID-19 is an abbreviation of coronavirus disease 2019	289	99.7
COVID-19 is caused by the SARS-CoV-2	272	93.8
A person infected with COVID-19 may have symptoms such as cough, fever and shortness of breath or no symptoms at all	288	99.3
SARS-CoV-2 allegedly came from the order Chiroptera (bats)	258	89.0
COVID-19 can be transmitted through droplets or aerosol transmission (liquid that comes out of the respiratory tract when coughing, sneezing or talking) in people who have symptoms or no symptoms at all	287	99.0
COVID-19 can be treated using antibiotics	77	26.6
COVID-19 can spread through airborne means (aerosol) in the community	86	29.7
Incubation period of COVID-19 is 3–7 days	97	33.4
The other way to prevent the spread of COVID-19 is to keep a distance of 1 m	19	6.6

COVID-19, coronavirus disease 2019; SARS-CoV-2, severe acute respiratory syndrome coronavirus 2.

Table 5 shows the indicator for assessing the attitudes of medical students towards COVID-19. It was found that the statement ‘implementation of the precautionary protocol that has been implemented does not interfere with my activities’ was agreed by 283 students (97.59%). Meanwhile, the statement that was least agreed was ‘I feel there is no need to wash my hands before touching my face’ (15 students disagreed with this statement).

**TABLE 5:** Distribution of respondents’ answers where respondents agreed with statements related to attitude towards COVID-19 (*n* = 290).

Statement	Frequency	Percentage (%)
1. I prefer to implement the preventive protocol that has been implemented by the government.	280	96.55
2. I prefer to be ethical when coughing or sneezing by covering my nose and mouth with the inner upper arm or tissue and also not littering with used tissues.	196	67.59
3. Implementation of the precautionary protocol that has been implemented does not interfere with my activities.	283	97.59
4. I believe that the spread of COVID-19 can be controlled by implementing physical distancing.	280	96.55
5. I will continue to regularly wash my hands even after this pandemic is over.	280	96,55
6. I feel the need to shake hands when I meet my friends.	24	8.28
7. I feel better going out of the house and to crowded places (more than 10 people) such as markets, coffee shops, etc. when I feel bored.	27	9.31
8. I don’t feel the need to use a mask when travelling.	23	7.93
9. I feel there is no need to wash my hands before touching my face.	15	5.17
10. I feel confident that COVID-19 infection can only be spread through people who have symptoms.	25	8.62

COVID-19, coronavirus disease 2019.

Table 6 shows the statement that is agreed by the majority of medical students in Aceh Province (248 students or 86.52%) in implementing health protocols, which is about avoiding crowds of people when going outside the house. Meanwhile, the statement that was less agreed upon is about not telling others if they have symptoms similar to COVID-19 (only 17 students or 5.86% agreed).

**TABLE 6:** Respondents’ answers related to preventive behaviour towards COVID-19 (*n* = 290).

Statement	Frequency	Percentage (%)
1. When I go outside the house, I maintain physical distancing from other people.	239	82.41
2. When I go outside the house, I avoid crowds of people.	248	85.52
3. When I meet other people, I refuse to shake hands.	169	58.28
4. When I travel, I choose to not use public transportation.	232	80.00
5. When I’m in a public area, I avoid touching objects in that area.	236	81.38
6. When I wash my hands, I ignore the recommendations given by the government (washing hands with soap for 20 s).	46	15.86
7. When I go out of the house, I don’t wear a mask.	18	6.21
8. When I do activities, I often touch my face without washing my hands first.	29	10.00
9. When I have symptoms similar to COVID-19, I don’t tell others.	17	5.86
10. When I receive goods/packages from outside, I don’t disinfect them before I bring them into the house.	62	21.38

COVID-19, coronavirus disease 2019.

### How knowledge influences medical students’ behaviour towards preventing COVID-19

It was found that there is a relationship between the knowledge and behaviour variables. The analysis shows that almost half of the medical students (46.6%) with just moderate knowledge have sufficient preventive behaviour too. A total of 44.5% of the medical students who have very good knowledge also show how good they are at preventing COVID-19. In this study, values of *p* < 0.05 and *r* = 0.214 were obtained, indicating a significant relationship between knowledge and prevention behaviour towards COVID-19 with a weak correlation strength. Therefore, it can be concluded that there is an influence on students’ knowledge about COVID-19 regarding their preventive behaviour.

### The influence of attitudes that medical students have on COVID-19 preventive behaviour

From the analysis, it was also found that there is a relationship between attitudes and preventive behaviour of medical students. This study shows that about 26 (9%) medical students with poor attitudes also have poor preventive behaviour. Likewise, medical students with good attitudes (73.1%) generally show good preventive behaviour. The values of *p* = 0.000 and *r* = 0.477 indicate a significant relationship between attitudes and behaviour with moderate correlation strength. Therefore, it can be interpreted that there is an influence on students’ attitudes to preventive behaviour against COVID-19.

## Discussion

This analytical observational study was conducted to investigate the knowledge, attitudes and preventive behaviour of medical students in Aceh Province regarding COVID-19. Because medical students are considered to have an important role in educating the public about health protocols, it is necessary for them to have a good knowledge and attitude towards COVID-19 as a role model to improve the surrounding community’s preventive behaviour. This study also links the relationship between variables, which allows comprehending how much one variable can influence another.

The measurement of knowledge about COVID-19 in medical students in Aceh Province was assessed using a questionnaire that the researchers had compiled by following validated expert opinion. This study found that most of the medical students in Aceh Province (54.1%) had good knowledge about COVID-19. This study is in line with research conducted in Jordan and Egypt showing that most medical or health students have good knowledge of COVID-19 (Alzoubi et al. 2020; Soltan, El-Zoghby & Salama 2020).

The studies in Jordan and Egypt found that almost all statements asked were answered correctly, with a prevalence of > 70% for correct answers. The research results in China also support the given statement, where out of a total of 4360 answers regarding the questions asked about knowledge, 3590 (82.34%) indicated the correct answer regarding COVID-19. In addition to the given study, research in Korea also showed that as many as 53.7% of research respondents had knowledge above average. It could be concluded that students’ knowledge of COVID-19 was good (Kim et al. 2020b; Peng et al. 2020).

The facts that medical students already knew about COVID-19 are detailed as follows:

### Definition of COVID-19

The definition of the abbreviation COVID-19 is Coronavirus Disease 2019 (Huang & Zhao 2020; Olum et al. 2020). This statement was asked in this study so that students do not provide wrong information about the meaning of COVID-19 itself. In this statement, only one respondent of the 290 respondents answered incorrectly.

### Virus that causes COVID-19

Regarding this statement, the researchers considered that there were still many people who thought that COVID-19 was caused by bacteria, germs or the same as the previous SARS COV virus. However, the WHO has determined that COVID-19 is caused by the SARS-COV-2 virus (Huang & Zhao 2020; Ouassou et al. 2020).

### Clinical manifestations of COVID-19

The main symptoms caused by COVID-19 (dry cough, fever, shortness of breath, does not cause symptoms at all) were asked in this study to assess how well medical students recognised the symptoms that could be caused by COVID-19, amongst others (Thevarajan, Buising & Cowie 2020; WHO 2020c). In knowing the main symptoms of COVID-19, it is hoped that medical students will become more aware of all symptoms that COVID-19 can cause.

### The origin of the cause of COVID-19

The origin of the cause of COVID-19 is still in the stage of further research, but the WHO has determined that the animal causing this virus is thought to be the bat in accordance with the morphological similarities obtained between the COVID-19 virus in humans and bats (Burki 2020; Cascella et al. 2020).

### Transmission of COVID-19

The transmission mechanism of COVID-19 needs to be known so that medical students can provide awareness to the public so that they can then protect themselves from being infected by the virus. It is known that this virus can spread through droplets (aerosol), namely fluids that come out of the human respiratory system such as when speaking, coughing, sneezing and others (Jayaweera et al. 2020; Li et al. 2020).

### The use of antibiotics in treating COVID-19

The use of antibiotics as drugs that can treat COVID-19 is considered wrong because COVID-19 is a virus, so it cannot be treated with antibiotics. The antibiotics are usually used to treat secondary infections that may occur in sufferers of COVID-19 (Kim & Walker 2020; Kronbichler et al. 2020).

### Incubation period for COVID-19

The incubation period for COVID-19 according to the WHO is 2–14 days. Regarding this statement, there are still many mistakes medical students make when answering statements related to the incubation period for COVID-19 (Backer, Klinkenberg & Wallinga 2020; Lauer et al. 2020). In this study, a total of 97 respondents (33.4%) answered incorrectly on this statement.

### Physical distancing

Maintaining distance from one another is considered to be an action that can help in breaking the chain of the spread of COVID-19. However, the distance determined by health protocols is actually not 1 m but must be more than 2 m between one person and another (Chu et al. 2020).

From the above statements, it can be found that medical students have good knowledge of COVID-19. Most likely, this happens because medical students have broader desires and access than other students. It should be observed that students are currently being encouraged by universities to be able to assist the government in implementing the protocols that have been given to the community. This is performed by encouraging students, especially medical students, to conduct internships with the COVID-19 theme, which are carried out simultaneously and in various regions. Hence, with this programme medical students are encouraged to be able to understand material about COVID-19 by understanding symptoms, prevention and more. Therefore, medical students must be able to build up a good knowledge of COVID-19.

Several factors can influence a person’s attitude, including personal experience, the influence of other people who are considered important, culture, mass media and educational institutions. Research conducted in Jordan and Korea shows that the respondents’ attitudes towards behaviour reflects the correct way to prevent COVID-19 by various means, namely washing hands, using alcohol, avoiding shaking hands, following precautionary ethics during coughing and sneezing or the other health protocols that can help control the COVID-19 disease (Alzoubi et al. 2020; Kim et al. 2020a).

The results of the present study were also supported by research in China. In a study of 876 students from 10 universities in China, it was found that from 4360 answers received, 3834 respondents (87.94%) chose proactive answers to statements given, thus they were considered to have a good attitude in assessing prevention behaviour against COVID-19. However, it is noteworthy that the researchers specified the sample to be medical students, and not only health students, as was performed in research in China (Peng et al. 2020; Wake 2020).

The following are the hesitant attitudes that the majority of medical students still had regarding the prevention of COVID-19: ‘I feel the need to shake hands when meeting my friends, I feel it is better to go out of the house and to a crowded place (more than 10 people) such as a market, the coffee shops and others if I feel bored, I don’t feel the need to use a mask when travelling, I don’t feel the need to wash my hands before touching my face, I feel sure that COVID-19 infection can only be spread through people who have symptoms’.

The present study shows that several factors still lead to doubts regarding COVID-19 for the majority of medical students in Aceh Province. Firstly, there are still many medical students who have doubts about the handshake when meeting friends. In contrast, according to the public’s protocol, they are advised to have minimal physical contact with each other in order avoid being exposed to the virus (Kucharski et al. 2020; IDAI COVID-19 Team 2020). Secondly, many medical students also have doubts about going to a crowded place such as a shopping mall, a coffee shop and others when they feel bored according to the government rules, these venues are categorised as forbidden places in the lockdown phases. However, it is hoped that the public will limit travelling to overcrowded places if it is not necessary because the virus can spread without causing overt symptoms in sufferers. Thirdly, some medical students have doubts about the need to wear masks when travelling. Meanwhile, it has been explained that in conditions such as these, the public must wear masks when travelling; this is because the current spread occurs through droplets, airborne elements (aerosol) and direct physical contact (Esposito et al. 2020; WHO 2020a). Fourthly, many students are still hesitant to limit touching their face without first washing their hands. In contrast, according to the government’s protocol, the community is expected to restrict self-touching of faces because the face is a sensitive area and is close to the initial transmission of the COVID-19 disease (Chu et al. 2020; Przekwas & Chen 2020). Finally, many students do not believe that COVID-19 can spread from asymptomatic people. At the same time, the WHO determined that this virus can be spread not only through people who have symptoms but also through people who have no symptoms. Therefore, people are urged to pay attention to health protocols before leaving their homes. Whilst travelling, one can exposed to this virus from persons even though they may be asymptomatic (Kim et al. 2020b; WHO 2020c).

This study also found that the majority of medical students behaved well in complying with the preventive protocols set by the Government through posters, flyers and mass media. It was found that 264 medical students (91.1%) carried out predetermined preventive behaviours more than adequately required such as wearing masks, washing hands as recommended, avoiding crowds, maintaining distance from one another, and others (Lauer et al. 2020).

Regarding the subject of behaviour in the questionnaire, the researcher gave positive and negative statements to assess the behaviour of the respondents. Naturally, this statement was related to the preventive behaviour against COVID-19 that has been established by the government. The type of behaviour obtained in the given statement also illustrates how respondents can behave in this pandemic situation, which is similar to some other research undertaken in Saudi Arabia and China. Those research cases show that some students have a sufficiently good level of behaviour in preventing the virus so that the publication of the health protocol is considered efficient in helping students to behave properly (Alzoubi et al. 2020; Peng et al. 2020).

This study also shows that the majority of medical students in Aceh Province performed several health protocols. Firstly, most medical students have carried out physical distancing when travelling out of the house or in a crowd. This physical distancing is considered to have the ability to help break the chain of the spread of COVID-19 because the main spread of this virus is through droplets (aerosol) so that it is hoped that the droplets released do not reach other people and the person is not subsequently exposed to the virus (Backer et al. 2020; Lauer et al. 2020). Secondly, the majority of medical students have avoided crowds when going out of the house. This is also considered effective in stopping the spread of COVID-19, as mentioned in the previous statement (CDC 2020). Thirdly, most medical students have also not travelled using public forms of transportation. Someone who leaves their house to travel is vulnerable to being exposed to this virus when talking, shaking hands, making direct physical contact or spreading the virus indirectly (when droplets are attached to passenger seats, door handles, helmets etc. when travelling by public transportation) (Rawat, Kumari & Saha 2020). Fourthly, medical students have also avoided touching items or objects in public places because they consider these items a transit point for this virus before it spreads indirectly (IDAI COVID-19 Team 2020). From the present study, it was found that medical students have good preventive behaviour against COVID-19. This may occur because medical students have easy access to information about prevention protocols and an obligation to educate the surrounding community. The Minister of Education and Culture of the Republic of Indonesia encourages medical students to be the second guard against COVID-19, by disseminating information and educating the public to comply with the preventive protocols.

In addition, this study found that the better the medical students’ knowledge of COVID-19, the better their good behaviour in preventing the virus, based on the Spearman’s correlation test, which was carried out with the results of *p* = 0.000 (*p* < 0.05) and the correlation coefficient value *r* = 0.213. These results indicate that there is a correlation between knowledge and prevention behaviour even though it has a weak correlation strength. This is also supported by research conducted at Mutah University in southern Jordan on health students and non-health students but in this study the obtained results were more specific to medical students so that the researchers could accurately determine how well medical students understood the COVID-19 virus (Alzoubi et al. 2020).

According to the analysis, the knowledge possessed by medical students is in line with the preventive behaviour. This is likely because of the knowledge possessed by students that has reached the application level, so that the knowledge they have will greatly influence their actions. Medical students are also motivated by the obligations to have an important role to help educate the public through information that is certainly easier to obtain at this time.

This study also found that the better the attitudes of medical students, better they would behave in complying with the preventive protocols set by the government, which was based on the Spearman’s correlation test carried out, within which the *p* = 0.000 (*p* < 0.05) and the correlation coefficient value *r* = 0.477 (moderate correlation) were found. These results indicate that there is a relationship between attitude and preventive behaviour which is significant. Furthermore, this research is in line with a survey conducted by Saefi et al. (2020), which shows that there is a significant relationship between COVID-19 prevention behaviour and the score of medical students’ attitudes towards COVID-19 (*p* = 0.000). This study also aims to provide data to be developed in further research (Saefi et al. 2020). In addition, other studies also state that preventive behaviour and attitudes have a significant relationship. This is evidenced by the results of the analysis using the Spearman’s correlation that was obtained (*p* < 0.05) (Salkind 2010).

From the present study, it was found that the attitudes of medical students were in line with their preventive behaviour against COVID-19. This may occur because students are accustomed to understanding health protocols and the obligations of medical students in educating the public to comply with established preventive protocols and are also supported by current internet facilities that allow medical students to instantaneously obtain more extensive information about COVID-19.

## Conclusion

This study found that most medical students have good knowledge, attitudes and behaviour towards COVID-19. This is shown by the good results obtained, exceeding 50% for knowledge and attitudes, and 46.6% of students who can behave well enough to comply with established health protocols. This research also shows that there is an influence of knowledge on preventive behaviour that will be carried out against COVID-19. Even though the strength of the relationship is weak, there are still bonds that show it is mutually influential. In addition to the influence shown by knowledge, this study also shows that there is an influence between the attitudes of students on their preventive behaviour, the same as before, but the effect of attitudes on behaviour is considered to have a stronger bond compared with the influence of knowledge on preventive behaviour.

It is very important to continue to improve the knowledge and attitudes possessed by students in order to be able to influence preventive behaviour that will be carried out by these students. Therefore, the increased understanding that students have of COVID-19 will also enhance the understanding carried out by the community, which in turn will make the spread of the COVID-19 disease that occurs more controllable.
